# Phyllosphere bacteria with antiquorum sensing and antibiofilm activities against fish pathogenic bacteria

**DOI:** 10.1186/s13104-023-06657-9

**Published:** 2024-01-02

**Authors:** Griselda Lukman, Diana Elizabeth Waturangi, Pande Gde Sasmita Julyantoro, Nurmaya Papuangan

**Affiliations:** 1 Department of Biotechnology, Faculty of Biotechnology, Atma Jaya Catholic University of Indonesia, Jalan Jenderal Sudirman 51, Jakarta, 12930 Indonesia; 2 Department of Aquatic Resources Management, Faculty of Marine Science and Fisheries, University of Udayana, Denpasar, Bali, 80361 Indonesia; 3 Department of Biology Education, Faculty of Teacher Training and Education, Khairun University, Ternate, 97728 Indonesia

**Keywords:** Antibiofilm, Aquaculture system, Fish pathogenic bacteria, Quorum quenching, Quorum sensing

## Abstract

**Objective:**

This research aims to quantify antiquorum sensing and antibiofilm activity of f phyllosphere bacteria against biofilm formed by pathogenic fish bacteria such as *Aeromonas hydrophila*, *Streptococcus agalactiae*, and *Vibrio harveyi*.

**Results:**

Antiquorum sensing assay using *Chromobacter violaceum* as indicator bacteria and antibiofilm assay showed six phyllosphere bacteria have antiquorum sensing and antibiofilm activities against tested bacteria. The highest inhibition and destruction activity was showed by metabolite of JB 3B and EJB 5 F against *A. hydrophila*, respectively. Determination using light microscope and scanning electron microscope performed decreaing in biomass of biofilm observed after treated with metabolite from phyllosphere bacteria.

**Supplementary Information:**

The online version contains supplementary material available at 10.1186/s13104-023-06657-9.

## Introduction

Indonesia is an archipelagic country with a total water area of around two-thirds of the territory of Indonesia. One of the immense potentials that Indonesia has is the development of the fisheries sector [1]. However, one of the biggest problems faced in the aquaculture industry in Indonesia is fish infectious diseases, which caused by the several fish pathogenic bacteria, can lead to the failure of fish farming This infectious diseases usually treated with antibiotics [2, 3].

*Aeromonas hydrophila*, *Streptococcus agalactiae*, and *Vibrio harveyi* are several aquaculture pathogenic bacteria that cause infectious diseases [4, 5]. Generally, antibiotics are used to treat bacterial infections in aquaculture. Prolonged use of antibiotics can cause resistance in fish pathogenic bacteria and pose a potential health risk to humans as consumers. The important things are several fish pathogenic bacteria are able to form biofilm matrix to be able to survive in aquaculture systems especially in the condition of environmental pressure [3]. Matrix of biofilm provides the bacteria a suitable environment andprotect bacteria from antibiotics, antimicobe as well asenvironmental pressure, it will help the bacteria to survive in the aquaculture environment [6]. The importance mechanism in forming biofilm is cell to cell communication which are quorum sensing. When the densities of the cell high it will start to regulate and express gene related with biofilm formation. Therefore one of the strategy to inhibit biofilm formation is through inhibition of quorum sensing [7].

The phyllosphere is an aerial surface of plants, which is a habitat for microorganisms. Infectious diseases in plants caused by pathogenic bacteria can be prevented by components produced from other phyllosphere bacteria. These bacteria reported capable inhibit quorum sensing as well as antibiofilm, the components, such as, lactonase, have anti-quorum sensing activity that can degrade signaling molecules from pathogenic bacteria before they can infect plants have been reported [8]. Therefore an exploration of these microbe need to be explored.

On the other hand, our previous study from phyllosphere bacteria on antibiofilm against *A. hydrophila*, *S. agalactiae*, and *V. harveyi* has been carried out, they found crude extracts (20 mg/mL) from phyllosphere bacteria showed antibiofilm activity, especially JB 3B and JB 20B. Therefore, phyllosphere bacteria have the potential to be applied in aquaculture systems to prevent and treat infectious diseases by pathogenic fish bacteria [7].

## Methods

### Bacterial cultivation

We used six phyllosphere bacteria isolates (JB 3B, JB 16B, JB 20B, JB 26B, JB 12 F, and EJB 5 F) were obtained from previous study by Juliana [9], which were isolated from leaf surface of *Psidium guajava* in Karanganyar, Jakarta. *Chromobacterium violaceum* wild type and *Chromobacterium violaceum* 026 were used as indicator bacteria. In addition, this study also used several fish pathogenic bacteria, namely *S. agalactiae* ATCC279956, while *A. hydrophila*, strain OF 83 (GenBank accession number MW624435.1) and *V. harveyi* isolated from infected shrimp were obtained from Health Aquatic Organism Laboratory, Department of Aquaculture, Faculty of Fisheries and Marine Sciences, Bogor Agricultural University.

The phyllosphere isolates streaked onto King’s B 10% (20 g Protease Peptone; 1.5 g K_2_HPO_4_; 1.5 g Mg_2_So_4_. 7H_2_O; 10 mL Glycerol; 10 g Agar Bacto; 1 L distilled water) then incubated at 28 °C for 48 h. *C. violaceum* wild type and *C. violaceum* 026 were streaked onto Luria Agar (LA) (Oxoid) then incubated 28 °C for 48 h. *A. hydrophila* was inoculated onto LA and incubated at 28 °C for 24 h. *S. agalactiae* was inoculated onto LA and incubated at 37 °C for 24 h. Meanwhile, *V. harveyi* was inoculated onto LA supplemented with 2% of NaCl (w/v) and incubated at 28 °C for 24 h.

### Production of supernatant

Each phyllosphere isolates were inoculated into 100 mL of Luria Broth (LB) (OXOID) then incubated at 28 °C for 48 h with 120 rpm agitation speed. Culture suspension was centrifuged at 5752 xg for 20 min. Cell-free supernatant was concentrated five times using vacuum oven at 50 °C. Then, concentrated supernatants kept at -20 °C [10].

### Detection of quorum quenching activity

*C. violaceum* as indicator strain was grown in LB and adjusted to 0.132 at 600 nm. As much as 100 µL of bacterial culture was streaked onto Brain Heart Infusion Agar (BHIA)(OXOID) using a continuous streak with a sterile cotton bud. Wells were formed with a sterile cork borer. After that, 100 µL of supernatants, streptomycin (10 mg/mL) used as a positive control, and DMSO (Dimethyl Sulfoxide) 1% (v/v) were used as a negative control and then pipetted into each well. Then plates were incubated at 28 °C for 24 h. This detection assay was performed in triplicates [7].

### Validation of quorum sensing inhibition

*C. violaceum* 026 mutant which could not produce acyl homoserine lactone, therefore do not produce violacein pigment was used as indicator strain was grown in Brain Heart Infusion Broth (BHIB) (OXOID) and adjusted to 0.1 at 540 nm. As much as 500 µL of bacterial culture and 500 µL of supernatant (1:1) were mixed in a microtube. Then, added with 1 µmol/mL N-Hexanoyl-1-Homoserine-Lactone (HHL). Mixture of bacterial culture and HHL were used as positive control. Meanwhile, negative control was used only *C. violaceum* 026. Microtubes were incubated at 28 °C for 24 h, then centrifuged at 5214 xg for 15 min. The supernatant was discarded, and then the pellet was mixed with 1 mL of DMSO 1% (v/v). After that, microtubes were centrifuged at 5214 xg for 15 min. The absorbance of supernatant was measured at 540 nm. This validation assay was performed in triplicates [11].

### Quantification of antibiofilm

This assays were divided into inhibition and destruction assay. For the antibiofilm assay on biofilm of *A. hydrophila*, all of supernatants except JB 20B were used due to the presence of antimicrobial activity. For the antibiofilm assay on biofilm of *V. harveyi*, all of supernatants except JB 16B due to the presence of antimicrobial activity and JB 20B were used. For the antibiofilm assay on biofilm of *S. agalactiae*, all supernatants except JB 20B and EJB 5 F were used due to their antimicrobial activity against this bacteria.

For the inhibition assay, 100 µL of bacterial culture with OD_600_ = 0.132 and 100 µL of supernatants were transferred into 96 wells microplate. *A. hydrophila* and *V. harveyi* were incubated at 28 °C, while *S. agalactiae* was incubated at 37 °C for 24 h. For destruction activity, 100 µL of each fish pathogenic bacteria was transferred into 96 wells microplate and then incubated at the same temperature as the inhibition assay. Then, 100 µL of supernatants were added to each well. Microplates were re-incubated with the same temperature overnight.

After incubation, media and planktonic cells were discarded. Each well was rinsed with sterile aquades. Before the cells were stained, adherent cells were allowed to air-dried for 30 min. After that, 200 µL of crystal violet 0.4% (w/v) was added to each well for 30 min. Then, the dye was discarded and rinsed with sterile aquades five times. Biofilm was allowed to air-dried for 30 min. A total of 200 µL of ethanol 96% was added to each well. The optical density of each suspension was measured at 595 nm. Sterile BHIB was used as blank. The formula for determining the percentage of inhibition [10]:

1 $$\% inhibition/destruction{\text{ }} = \frac{{OD{\text{ }}Control - OD{\text{ }}Sample}}{{OD{\text{ }}Control}} \times 100$$

### Microscopic observation of biofilm

Biofilms were observed using a light microscope. Furthermore, the selected supernatants, namely JB 16B, was further analyzed by SEM and EDS observations. Selected fish pathogenic bacteria were grown in BHIB and adjusted to 0.132 at 600 nm. Then biofilm was allowed to grow in a sterile cover glass for 24 h. A total of 100 µL of selected supernatant was added to the cover glass, then incubated for another 24 h.

For observation using light microscope, cover glass was rinsed using sterile aquades and stained with crystal violet for 15 min. After that, cover glass was rinsed using sterile aquades and observed with microscope.

For observation using Scanning Electron Microscopy (SEM), cover glass fixated in glutaraldehyde 2% at 4 °C for 24 h. Then, cover glass was dehydrated with alcohol 30% for 15 min, alcohol 50% for 15 min, alcohol 70% for 15 min, and alcohol 96% for 15 min. The cover glass was dried at 37 °C for 10 min. After that, the specimen was coated with gold (Au), and SEM and Energy Dispersive X-Ray Spectroscopy (EDS) was used to examine the surface of biofilm structure with magnification at 1000X, 2000X, and 5000X [12, 13].

### Identification of the phyllosphere isolates

Molecular identification was carried out on five phyllosphere isolates, namely JB 3B, JB 20B, JB 26B, JB 12F, and EJB 5F. DNA genomic extraction was done using Zymobiomics minirep kit. Furthermore, 16S rRNA gene sequence was amplified using 63F (5’*-*CAG GCC TAA CAC ATG CAA GTC − 3‘) and 1387R (5′-GGG CGG WGT GTA CAA GGC-3’) primer [14]. As much as 1 µL of the DNA template was added into a 12.5 µL of the GoTaq, 1 µL of each primer, and 9.5 µL ddH_2_O for PCR reaction mixture. The DNA was amplified using the following steps: pre-denaturation at 94 °C for 5 min, 30 cycles of 30 s denaturation at 95 °C, annealing at 55 °C for 30 s, elongation at 72 °C for 30 s and the post elongation 72 °C for 7 min. After that, PCR product was separated using 1.5% agarose gel electrophoresis for 45 min with 80 V, then visualized [15]. The results were sequenced by Genetika Science and the results were submitted to GenBank.

## Results

### Detection of quorum quenching activity

From six metabolite of phyllosphere isolates, two of them had positive results, namely JB 16B and EJB 5 F. Both isolates showed inhibition of violaein pigment production from *C. violaceum* which indicated by formation of a transparant zone around the wells (supplementary Fig. 1).

### Validation of quorum sensing inhibition

**Fig. 1:**
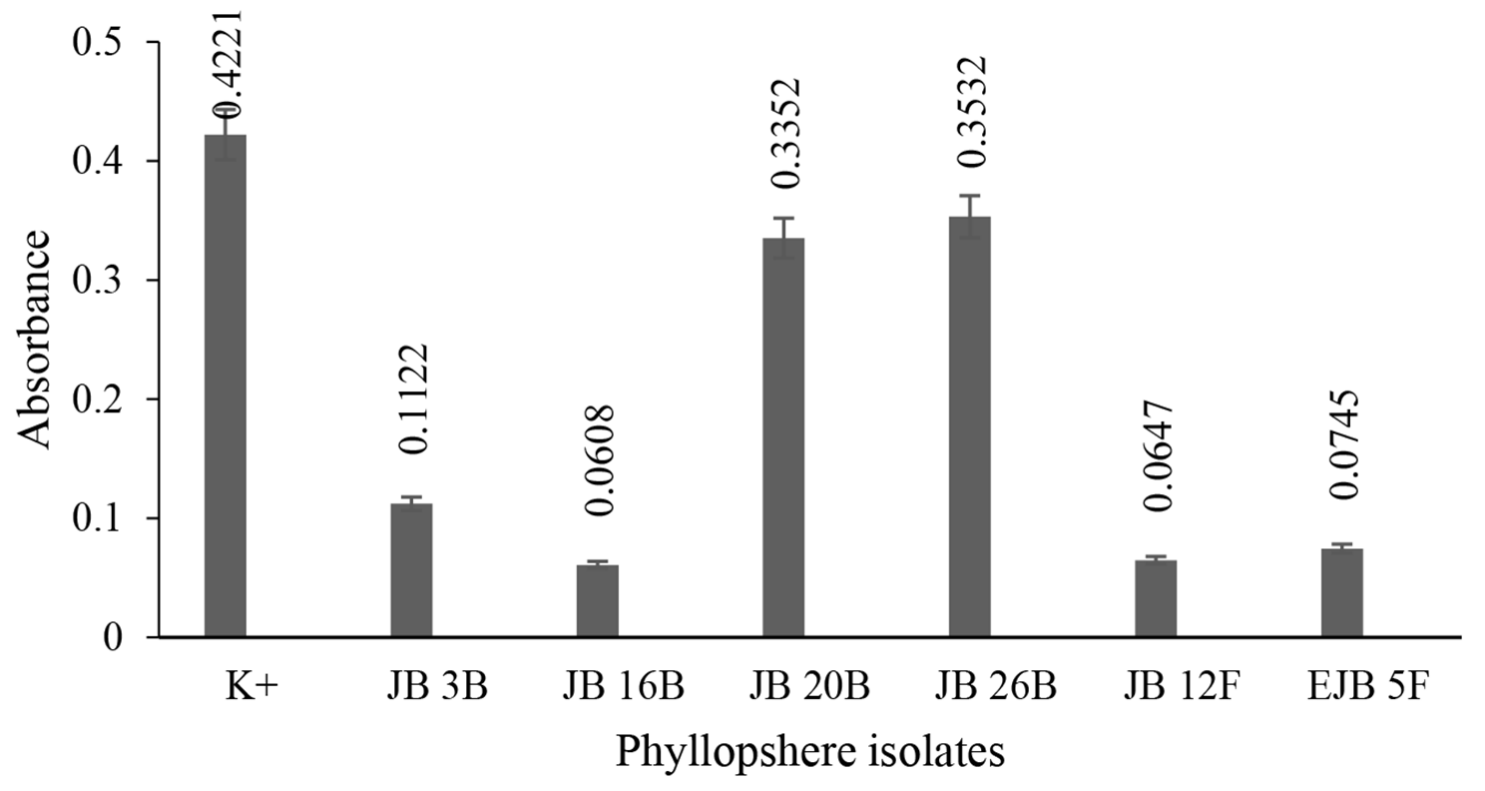
Validation of quorum sensing inhibition against *C. violaceum* 026 K+: positive control

All metabolite of phyllosphere had antiquorum sensing activity with indicated by lower absorbance after treatment. The results showed that metabolite from isolate JB 16B had the highest quorum sensing inhibition against *C. violaceum* 026 by 0.3613 absorbance difference (Fig. 1).

### Quantification of antibiofilm

**Fig. 2:**
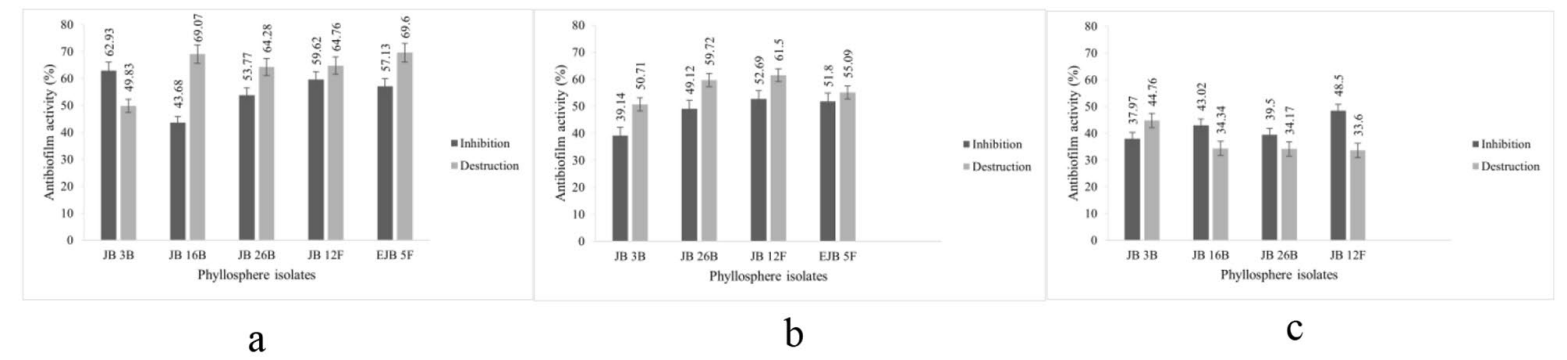
Antibiofilm activity against **(a)**  *A. hydrophila*, **(b)**  *V. harveyi*, **(c)**  *S. agalactiae*

All of the metabolite could inhibit biofilm formation and destruct mature biofilm. JB 3B and EJB 5 F performed the highest inhibition activity (62.93%) and highest destruction activity (69.6%), against biofilm of *A. hydrophila*, respectively. JB 12 F had the highest inhibition activity (52.69%) and highest destruction activity (61.5%), against biofilm of *V. harveyi*, respectively. Meanwhile, JB 12 F and JB 3B had the highest inhibition activity (48.5%) and highest destruction activity (44.76%), against biofilm of *S. agalactiae*, respectively (Fig. 2).

### Microscopic observation of biofilm and EDS

**Fig. 3:**
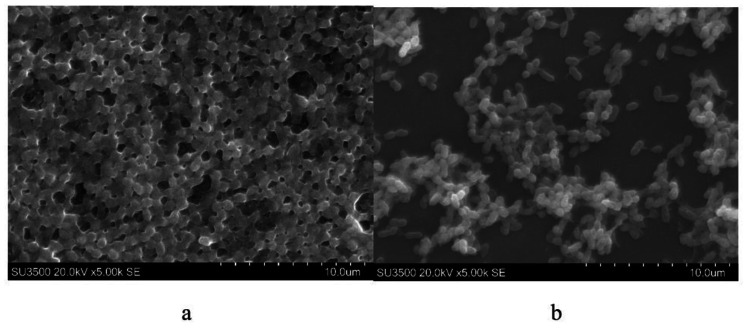
SEM determination of **(a)** biofilm of *A. hydrophila*  **(b)** destruction activity by JB 16B metabolite on biofilm of *A. hydrophila*

Microscopic determination was carried out using selected isolate based on antibiofilm activity. The structure of biofilm after treatment was observed using light microscope (supplementary Figs. 2 and 3) and these result was confirmed by using SEM observation (Fig. 3). Element contain of both pathogenic biofilm was characterized using EDS (supplementaray Table 1).

### Identification of the phyllosphere isolates

The five phyllosphere isolates were identified by using DNA sequencing of 16 S rRNA gene. It was found that all of five isolates showed similarities above 85% with their closest relatives. The results had been submitted to GenBank with the accession number (supplementary Table 2).

## Discussion

Fish infected by pathogenic bacteria is one of the problems faced by the aquaculture industry which can lead to the failure of fish farming and cause losses. Generally, this problem is resolved with the use of antibiotics. Biofilm is one of the abilities possessed by fish pathogenic bacteria to survive in aquaculture systems. However, cells in biofilm became more resistant to antibiotics than planktonic cells [3, 16]. In response to bacterial cell population density, communication between bacteria is regulated by quorum sensing, such as mediating gene expression for biofilm formation [17]. Inhibition of quorum sensing, commonly known as quorum quenching, can be used as an alternative way to inhibit biofilm formation.

Two of the six metabolite from phyllosphere isolates, namely JB 16B and EJB 5 F, had quorum quenching activity against *C. violaceum* wild type as indicator bacteria. *C. violaceum* is a bacterium that can produce violacein pigment which is regulated by quorum sensing by using autoinducer C6-AHL. Both metabolite contained bioactive components that could inhibit the production of violacein from *C. violaceum* wild-type [18]. Meanwhile, the other four metabolite did not appear to have quorum quenching activity. This could be due to the low concentration of bioactive in the metabolite.

All of the metabolite from the phyllosphere have quorum quenching activity against *C. violaceum* 026, which is a mutant of *C. violaceum* wild-type that cannot produce violacein due to the insertion of double transposon Tn5. However, these mutants can still recognize AHL and produce violacein [19]. The lower the absorbance indicates lower violacein production due to the presence of bioactive components that act as quorum sensing inhibitors. The quorum sensing mechanism can be inhibited by quorum sensing inhibitors through direct inhibition of signalling molecule synthesis, signal molecule transport inhibition, degradation of signal molecules, and competitive inhibition of signal molecules and receptors [7, 20].

Metabolite of phyllosphere showed antibiofilm activity but their activities varied greatly and specific between fish pathogens. However, the antibiofilm activity of the six metabolite against *S. agalactiae* performed as the lowest compared to the other two pathogens. Each phyllosphere isolates may have different bioactive compounds that have different antibiofilm mechanisms against pathogenic bacteria. AHL-lactonase is an enzyme produced by phyllosphere bacteria that could cleave the lactone ring in AHL where AHL is used by Gram-negative bacteria such as *A. hydrophila* and *V. harveyi* [7, 21]. Each pathogen also has a different EPS component which can be degraded by different bioactive components, for example, enzymes such as proteases, glycoside hydrolases, and deoxyribonucleases. In addition, biofilm destruction may also be due to the presence of other molecules that can induce biofilm destruction [10, 22].

In addition, compared to studies conducted by Nathalia and Waturangi [7], it was found that all of the metabolite of phyllosphere bacteria had higher antibiofilm activity compare with crude extracts against biofilm of *A. hydrophila*. This may be due to differences in concentration and bioactive components between the supernatant and crude extract. There may be less polar bioactive components that are wasted when extracted using ethyl acetate where these components are the main compounds that act as antibiofilm and are specific to *A. hydrophila.*On previous studies, the formations of fish pathogenic biofilms can be inhibited by phyllosphere bacterial extracts. Phyllosphere bacteria receive pressure, such as limited nutrition and environmental conditions in their habitat on the leaf surface. These bacteria have the ability to produce metabolites to compete with each other to protect themselves. Metabolites from phyllosphere bacteria could have competencies to inhibit production of signal molecule that contributes in forming biofilm. Pili formed by *S. agalactiae* is contributed the most in biofilm formation. On *A. hydrophila*, metabolites could disturb AI-1 system that induce biofilm production or flagella formation that also plays role in biofilm formation. Meanwhile on *V. harveyi*, metabolites from phyllosphere bacteria extracts could disturb AHL-mediated QS mechanism that control biofilm formation [23].

Selected biomass of pathogens were observed using light microscopy and confirmed by using SEM observation. From the results of observations, it was seen that the biofilm from both the inhibitory and destructive activities decreased after being treated with the metabolite from phyllosphere. Inhibition of biofilm formation showed by the less cell biomass formed than the control. Meanwhile, destruction of formed biofilm showed by the damaged biofilm structure and not as dense as the control.

C, O and N had the highest total weight compared to other elements from both biofilm of pathogens. Those elements come from the main components of the biofilm matrix, namely polysaccharides and proteins. Inorganic elements such as Al, S, Na, Cl and P, contributing to forming biofilm structure and adhesion to surfaces can be found from both pathogens. The presence of P elements was detected from eDNA, while Ca and Mg were detected from cytoplasmic electrolytes. After treated with metabolites, Fe can be found from both biofilm, which can indicate the death of *V. harveyi* cells where the dead cells will release small amounts of FeDestruction of cell biomass is caused by a decrease in organic elements such as C, N, S, P and O which are the main and essential components of EPS. The decrease in cell biomass also caused by the weakening of the biofilm attachment to the surface due to a decrease in inorganic elements such as Ca, Mg, P, Al, and Si. Differences in distribution, components, and total weight could be due to the non-homogeneous surface of the sample and influenced by the adsorption capacity, temperature, and pH of the biofilm. In addition, it can be caused by other components from the metabolite sample [24–27].

One of the six phyllosphere isolates has been sequenced by a previously unpublished study, namely, isolate JB 16B which has similarities with *Proteus myxofaciens* [9]. The other five phyllosphere isolates were also sequenced. The results showed that two of the sequenced isolates, JB 3B (OM763955) and JB 12 F (OM914883) had similarities to *Pseudomonas fluorescens*. Meanwhile, JB 20B (ON171240), JB 26B (OM772761), and EJB 5 F (OM914981) showed similarities to *Proteus myxofaciens, Pseudomonas stutzeri*, and *Bacillus subtilis*, respectively. *Pseudomonas* is one of the genera commonly found in the phyllosphere environment [8].

It was reported that *Pseudomonas fluorescens* produces exopolysaccharides and pectinase which have antibiofilm activity. Exopolysaccharides can inhibit biofilm formation by suppressing eDNA production [28]. Meanwhile, the pectinase enzyme works as an antibiofilm agent by degrading the structure of EPS [29]. *Bacillus subtilis* has also been found to produce antibiofilm agents such as proteins and exopolysaccharides [21]. Surfactin, fengycin, and iturin are lipopeptides produced by *Bacillus subtilis* and act as anti-adhesive agents in inhibiting biofilm formation by changing the hydrophobicity of the cell surface. From another study, *Bacillus subtilis* also produces alpha-amylase that can destroy mature biofilms by hydrolyzing EPS. *Pseudomonas fluorescens* also produces psedudofactin II, a lipoprotein that has anti-adhesive activity [30, 31].

## Conclusion

The six phyllosphere bacteria have antiquorum sensing and antibiofilm activities for both inhibition and destruction of biofilms. Antibiofilm activity of the metabolite showed varying results in the three fish pathogenic bacteria. The bioactive components in the metabolite work specifically on certain bacteria as antibiofilm agents. The decrease in cell biomass in the biofilm was also observed by light microscopy and SEM observation. Half of the phyllosphere isolates used in this study, showed high similarities to *Pseudomonas*. Therefore, the metabolite from phyllosphere bacteria showed potential activities to be applied in the aquaculture industries. However, further studies are needed to identify the bioactive components and their antibiofilm mechanisms.

### Limitations

This research uses only a few fish pathogenic bacteria which represent common pathogens that cause infection in fish. The bioactive components of the phyllosphere metabolite and their antibiofilm mechanisms have not been identified, therefore further investigation is needed. The toxicity of the metabolite to fish in aquaculture systems also needs to be explored.

### Electronic supplementary material

Below is the link to the electronic supplementary material.

Supplementary Material 1

## Data Availability

All DNA sequencing data deposited in the Genbank publicly with accession number for isolate JB 3B with the accession number OM763955, JB 12 F (OM914883), isolate JB 20B (ON171240), JB 26B (OM772761), and isolate EJB 5 F with the accession number OM914981.

## Abbreviations

EPS: extracellular polymeric substances
AHL: Acyl homoserine lactone
