# Supplementary material for: Phyllosphere bacteria with antiquorum sensing and antibiofilm activities against fish pathogenic bacteria
Source: BMC Res Notes. 2024 Jan 2;17:5. doi: 10.1186/s13104-023-06657-9 (PMC10759618; doi:10.1186/s13104-023-06657-9)
Supplement: Supplementary file 1 — Supplementary Material 1 [file 13104_2023_6657_MOESM1_ESM.docx]

**Supplementary**


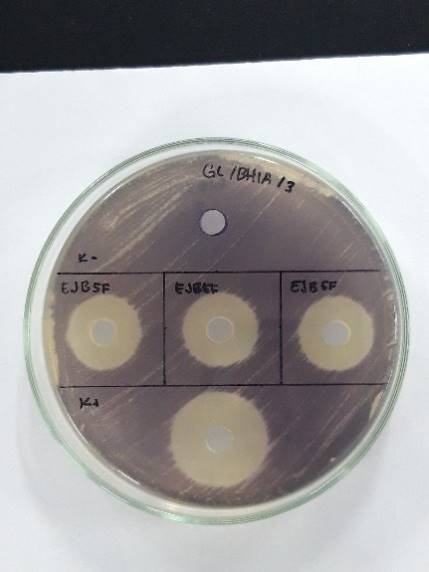

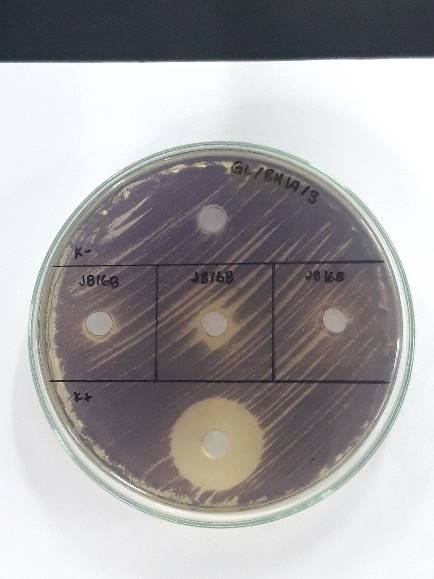


Supplementary Figure 1 Quorum quenching activity against *Chromobacterium violaceum* by (a) JB 16B supernatant, (b) EJB 5F supernatant


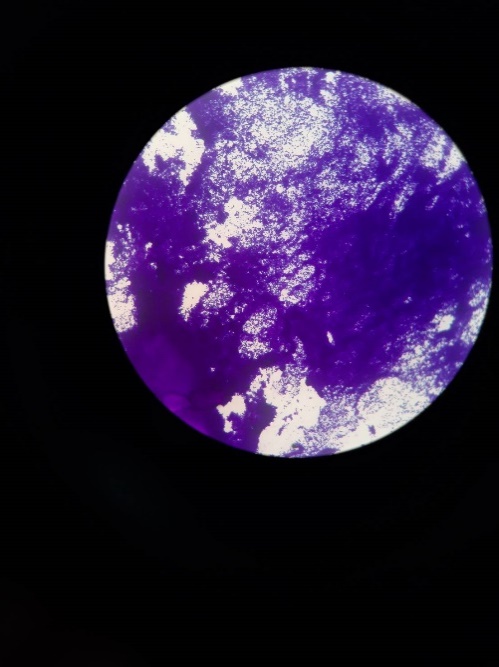

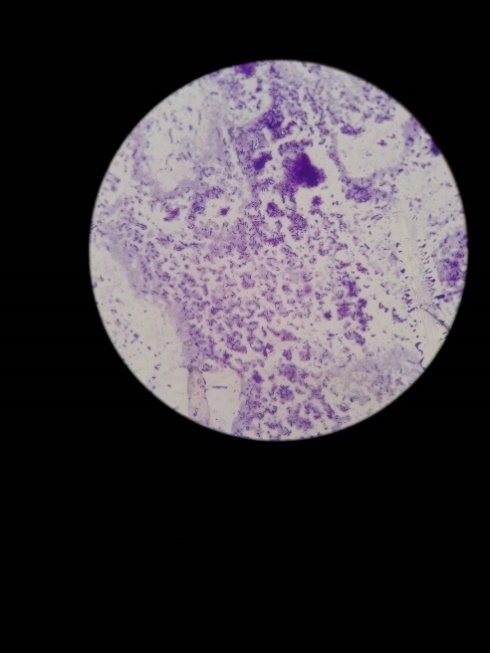


b

a


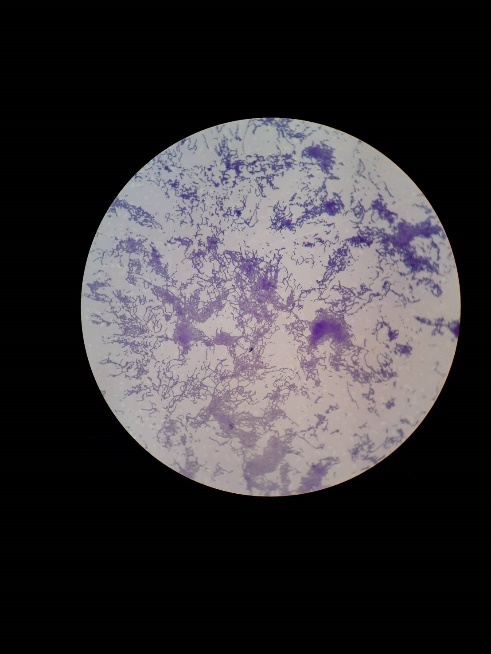

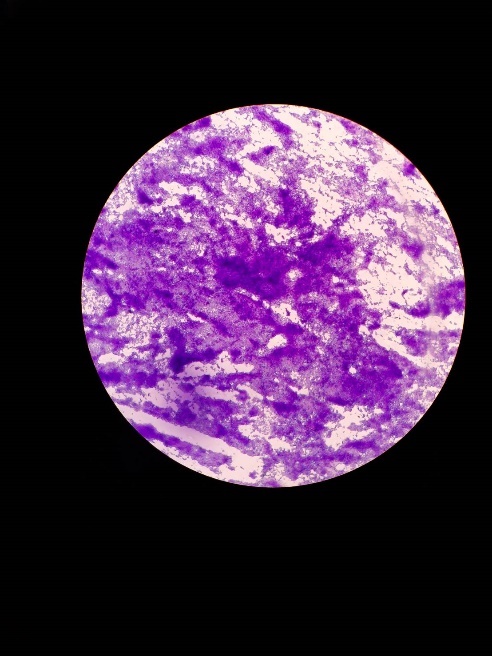


d

c

Supplementary Figure 2 Inhibition activity on fish pathogenic bacteria using light microscope (a) biofilm of *A. hydrophila* (control positive) (b) biofilm of *A. hydrophila* treated by supernatant of JB 3B (c) biofilm of *S. agalactiae* (control positive) (d) biofilm of *S. agalactiae* treated by supernatant of JB 12F


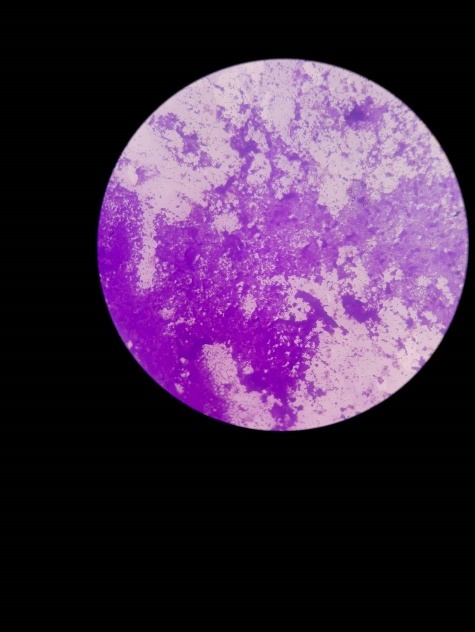

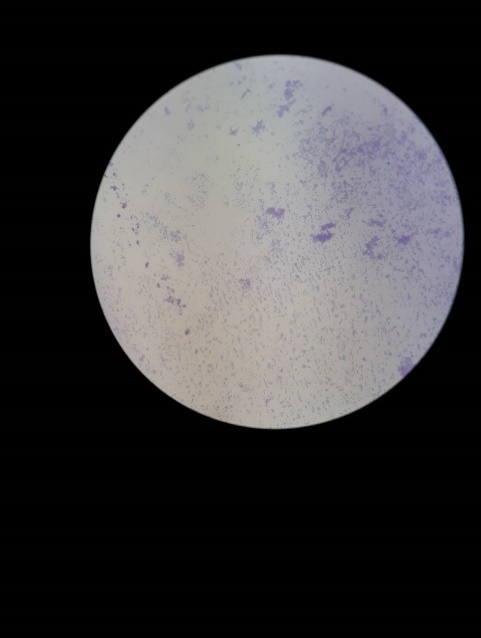

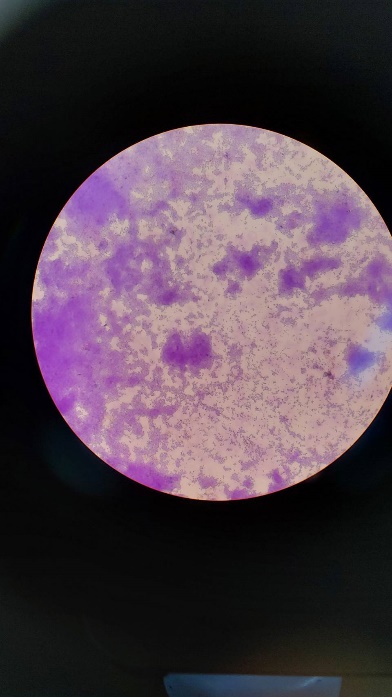


c

b

a


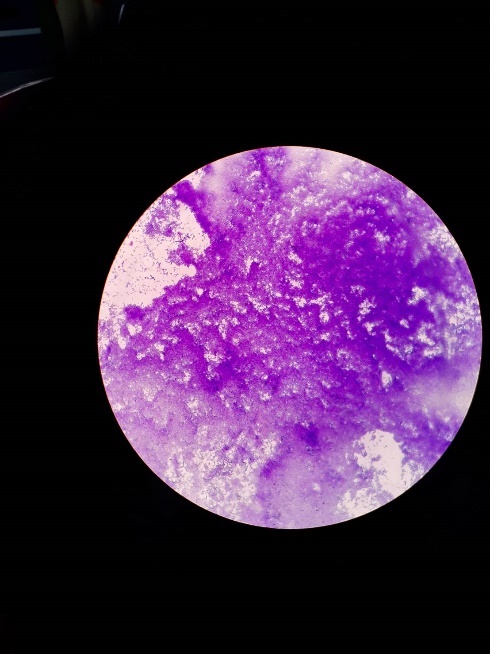

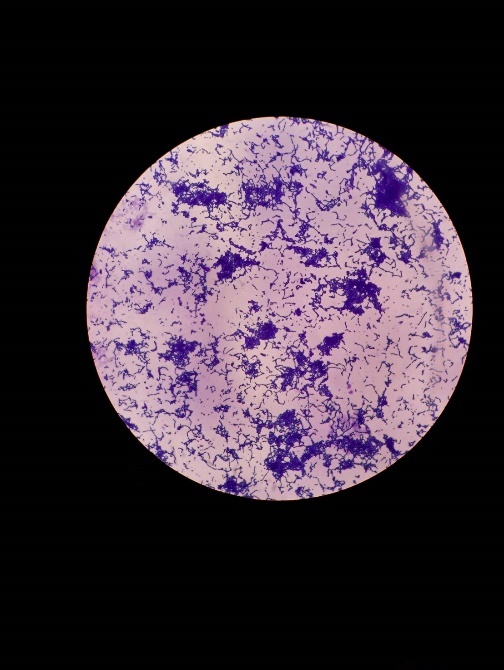


d

e

Supplementary Figure 3. Destruction activity on fish pathogenic bacteria using light microscope (a) biofilm of *A. hydrophila* (control positive) (b) biofilm of *A. hydrophila* treated by supernatant of JB 16B (c) biofilm of *A. hydrophila* treated by supernatant of EJB 5F (d) biofilm of *S. agalactiae* (control positive) (e) biofilm of *S. agalactiae* treated by supernatant of JB 3B

Supplementary Table 1 Total weight of biofilm element

| Element | A. hydrophila (%) | A. hydrophila  +  JB 16B (%) | V. harveyi (%) | V. harveyi  +  JB 16B (%) |
| --- | --- | --- | --- | --- |
| C | 49.16 % | 38.97% | 29.72 % | 36.87 % |
| N | 10.08 % | 6.31% | 5.53 % | 7.28 % |
| O | 18.77 % | 32.88% | 36.49 % | 34.91 % |
| Na | 2.35 % | 3.71% | 4.16 % | 3.90 % |
| Mg | 0.93 % | 1.00% | 1.25 % | 0.97 % |
| Al | 0.18 % | 0.21% | 0.25 % | 0.18 % |
| Si | 15.19 % | 14.49% | 19.17 % | 13.80 % |
| P | 0.28% | - | 0.85 % | 0.18 % |
| S | 0.12 % | 0.06% | - | 0.09 % |
| K | 0.12 % | 0.11% | 0.15 % | 0.09 % |
| Ca | 2.82 % | 2.26% | 3.13 % | 2.16 % |
| Fe | - | 0.06 % | - | 0.06 % |

Supplementary Table 2 DNA sequencing of phyllosphere isolates

| Isolate | Identification | % Similarity | GenBank accession number |
| --- | --- | --- | --- |
| JB 3B | *Pseudomonas fluorescens* strain UTB_111 | 92.23% | OM763955 |
| JB 20B | *Proteus myxofaciens* strain BMPM | 97.80% | ON171240 |
| JB 26B | *Pseudomonas stutzeri* strain FN9 | 99.36% | OM772761 |
| JB 12F | *Pseudomonas fluorescens* strain EB276 | 99.44% | OM914883 |
| EJB 5F | *Bacillus subtilis* strain T4 | 89.66% | OM914981 |
